# Microwave‐assisted extraction and ultrasound‐assisted extraction for recovering carotenoids from Gac peel and their effects on antioxidant capacity of the extracts

**DOI:** 10.1002/fsn3.546

**Published:** 2017-11-20

**Authors:** Hoang V. Chuyen, Minh H. Nguyen, Paul D. Roach, John B. Golding, Sophie E. Parks

**Affiliations:** ^1^ School of Environmental and Life Sciences University of Newcastle Ourimbah NSW Australia; ^2^ Faculty of Agriculture and Forestry Tay Nguyen University Buon Ma Thuot Daklak, Vietnam; ^3^ School of Science and Health Western Sydney University Penrith NSW Australia; ^4^ NSW Department of Primary Industries Ourimbah NSW Australia

**Keywords:** antioxidant, carotenoid, Gac peel, microwave, ultrasound

## Abstract

The peel of Gac fruit (*Momordica cochinchinensis* Spreng.) contains high levels of bioactive compounds, especially carotenoids which possess significant antioxidant capacities. However, the peel of Gac is regarded as a waste from the production of carotenoid‐rich oil from Gac fruit. In this study, carotenoids of Gac peel were extracted by microwave‐assisted extraction (MAE) and ultrasound‐assisted extraction (UAE) using ethyl acetate as extraction solvent. The effect of extraction time and different levels of microwave and ultrasonic powers on the yield of total carotenoid and antioxidant capacity of the extracts were investigated. The results showed that an extraction at 120 W for 25 min and an extraction at 200 W for 80 min were the most effective for MAE and UAE of the Gac peel samples, respectively. The maximum carotenoid and antioxidant capacity yields of UAE were significantly higher than those of the MAE. The antioxidant capacity of extract obtained by the UAE was also significantly higher that of the conventional extraction using the same ratio of solvent to material. The results showed that both MAE and UAE could be used to reduce the extraction time significantly in comparison with conventional extraction of Gac peel while still obtained good extraction efficiencies. Thus, MAE and UAE are recommended for the improvement of carotenoid and antioxidant capacity extraction from Gac peel.

## 1 INTRODUCTION

Gac fruit (*Momordica cochinchinensis* Spreng.) contains very high levels of carotenoids which includes lycopene and β‐carotene (Ishida, Turner, Chapman, & Mckeon, 2004; Vuong, Franke, Custer, & Murphy, 2006). The major commercial products from Gac fruit are oil and dried powder that are manufactured from the seed membrane (aril) of the fruit (Chuyen, Nguyen, Roach, Golding, & Parks, 2015; Kha, Nguyen, Roach, Parks, & Stathopoulos, 2013). Gac peel, which constitutes up to 15% of fruit weight, is considered as a waste in the manufacturing of the commercial products from Gac fruit. However, studies on Gac peel have showed that Gac peel contains high levels of carotenoids including β‐carotene, lycopene and lutein which possess significant antioxidant capacities (Chuyen, Roach, Golding, Parks, & Nguyen, 2017b; Kubola & Siriamornpun, 2011). β‐carotene is well‐known as a precursor to vitamin A, while lycopene and lutein have been reported as beneficial bioactive compounds for human health due to their antioxidant, anticancer and macular‐protective activities (Bernstein et al., 2016; Bhuvaneswari & Nagini, 2005; Vuong, Dueker, & Murphy, 2002). Thus if the carotenoids in Gac peel are recovered effectively, the peel may be a potential source of natural carotenoids for food, cosmetic and medicinal uses.

Our studies on the conventional extraction of bioactive compounds from Gac peel showed that carotenoids and antioxidant capacity from the peel can be efficiently extracted using organic solvents (Chuyen, Roach, Golding, Parks, & Nguyen, 2017c; Chuyen, Tran, et al., 2017). However, conventional methods require large volumes of solvents, high energy use and long extraction times for an efficient extraction of bioactive compounds. Recently, many advanced techniques for the extraction of bioactive compounds have been investigated to improve the extraction efficiency and overcome the disadvantages of conventional extractions. Among the newly developed techniques, microwave‐assisted extraction (MAE) and ultrasound‐assisted extraction (UAE) have been regarded as two of the most practical methods for the industrial scale due to the availability of equipment, the convenient operation and the high extraction efficiency (Wani, Bishnoi, & Kumar, 2016).

The MAE is based on the assistance of electromagnetic radiation with frequencies from 0.3 to 300 GHz, which induce heat inside the material via dipolar rotation and ionic conduction of the molecules (Camel, 2001). The activation of these molecules and the heat generated in this process may weaken or break the cell walls thereby the bioactive compounds can be released more easily from material matrix to the extraction solvents (Kaufmann & Christen, 2002). In another extraction technique, UAE improves the mass transfer of the extraction process by generating cavitation within the material. When the cavitation bubbles are produced and collapsed, the cell walls of the material will be destructed and the release of the solutes is promoted (Toma, Vinatoru, Paniwnyk, & Mason, 2001).

Previous studies have shown that the applications of MAE and UAE in carotenoid extraction can enhance efficiency, reduce solvent amount and save the extraction time compared with the conventional extraction methods. For example, the extraction time for carotenoids from carrots and algae was significantly reduced using continuous and intermittent microwave radiations (Hiranvarachat, Devahastin, Chiewchan, & Vijaya Raghavan, 2013; Pasquet et al., 2011). The UAE of lycopene from tomato waste has shown to occur with shorter extraction times, lower temperatures and smaller solvent volumes with higher extraction yields compared to the conventional extractions (Kumcuoglu, Yilmaz, & Tavman, 2014). These studies suggest that extraction of carotenoids from Gac peel may be improved with the assistance of microwave and ultrasound.

In this study, different power levels of microwave radiation and ultrasound and extraction times were investigated for enhancing the extractability of carotenoids from Gac peel. The effects of these parameters on antioxidant capacity of the extracts from the peel were also determined.

## 2 MATERIALS AND METHODS

### 2.1 Chemicals

Ethyl acetate and methanol were purchased from Merck Millipore (Bayswater, VIC, Australia). β‐carotene, Trolox standards, potassium persulfate and ABTS (2,2′‐azino‐bis(3‐ ethylbenzothiazoline‐6‐sulfonic acid) diammonium) were purchased from Sigma‐Aldrich Pty Ltd. (Castle Hill, NSW, Australia).

### 2.2 Material

Gac fruits at fully ripe stage were harvested at Wootton, NSW and transported to the laboratories at Central Coast campus of the University of Newcastle, Australia. The peel of the fruits was separated by a knife and dried to a moisture content of 4 ± 0.2%. The dried peel was then ground, mixed into a uniform lot and sieved by different size meshes. The ground peel with particle size of 250–500 μm was selected and stored in vacuum sealed bags in a freezer at −18°C in the dark until the extraction.

**Figure 1:**
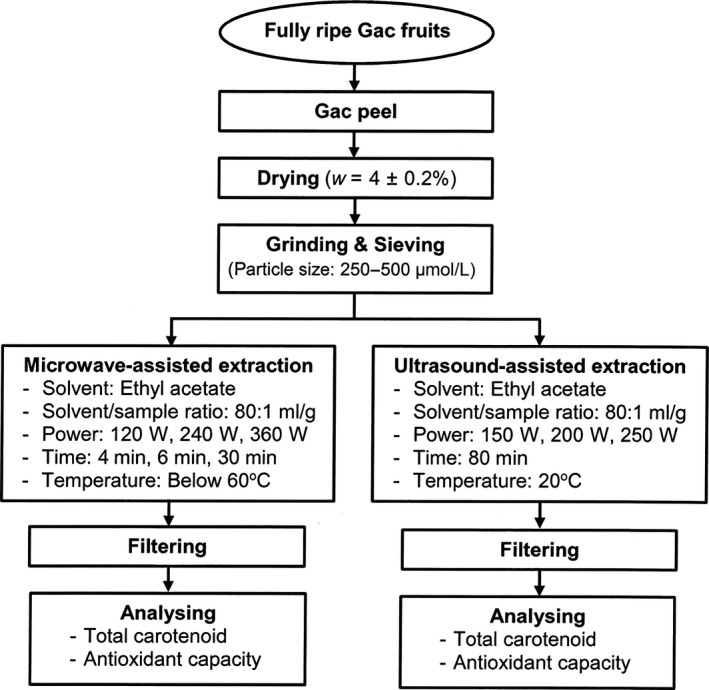
Preparation of Gac peel sample and experimental design

The diagram of sample preparation and experimental design is presented in Figure 1.

### 2.3 Experimental design

#### 2.3.1 Microwave‐assisted extraction (MAE)

A quantity of 0.5 gram of the dried Gac peel was extracted with 40 ml of ethyl acetate in a conical flask that was covered by glass fiber. The extraction was then carried out with a microwave oven (Sharp Carousel, Abeno‐ku, Osaka, Japan) that was placed in a fume hood for the ventilation of the evaporated solvent. An intermittent microwave radiation with 30 s of heating (“on”) and 30 s of non‐heating (“off”) alternatively was applied to avoid overheating of the extraction mixture. The extraction processes were terminated when the temperature reached 60°C. Three power levels (120, 240, and 360 W) were investigated for the extraction of carotenoids and antioxidant capacity of the extract.

The temperature of the extract was measured every minute using a digital thermometer (ThermoFisher Scientific, North Ryde, NSW, Australia). Following each minute of the extraction, the liquid phase was separated and filtered with a 0.45 μm cellulose syringe filter (Phenomenex Australia Pty. Ltd., NSW, Australia) to determine the total carotenoid content and antioxidant capacity.

#### 2.3.2 Ultrasound‐assisted extraction (UAE)

0.5 gram of the dried Gac peel was extracted with 40 ml of ethyl acetate in a conical flask. The flask was then covered by glass fiber and placed in an ultrasonic bath (Soniclean 1000HD, Soniclean Pty Ltd, Thebarton, SA, Australia) for the UAE extraction of carotenoids and antioxidant capacity from Gac peel. The extraction was carried out at power levels of 150, 200, and 250 W with an ultrasonic frequency of 43.2 kHz until the yield of total carotenoid and antioxidant capacity of the extract plateaued. The temperature was maintained at 20 ± 1°C throughout the extraction process by adding cold water to the ultrasonic bath. To examine the extraction yields of carotenoids and antioxidant capacity, the liquid phase was separated and filtered with a 0.45 μm cellulose syringe filter for further analysis.

#### 2.3.3 Measurement of absorbed microwave and ultrasonic powers

The microwave and ultrasonic powers absorbed by a mass unit of the extraction solution were determined using the following equation (Hiranvarachat & Devahastin, 2014; Ordóñez‐Santos, Pinzón‐Zarate, & González‐Salcedo, 2015):

$$P=C_{p}\frac{\Delta T}{t}$$

where P is the power absorbed by a mass unit of the extraction solution (W/g), C_p_ is the specific heat capacity (J/g.^o^C), ΔT is the temperature increase by the irradiation process (^o^C) and t is the irradiation time (s).

In this study, the absorbed microwave power at 120, 240 and 360W was determined as 0.32, 0.69 and 0.83 W/g, respectively. The ultrasonic power absorbed at 150, 200 and 250W was 0.75 × 10^−2^, 1.16 × 10^−2^ and 1.26 × 10^−2^ (W/g), respectively.

### 2.4 Determination of total carotenoid content

The absorbance at 450 nm of the extracts from Gac peel or standard solutions was determined using a Cary 50 Bio UV‐Visible spectrophotometer (Varian Australia Pty. Ltd., Mulgrave, VIC, Australia). The total carotenoid content of the extracts was expressed as mg β‐carotene equivalent/100 g dry weight (DW) based on the standard curve of β‐carotene in ethyl acetate.

### 2.5 Determination of antioxidant activity

To evaluate the antioxidant capacity of a bioactive compound or an extract, different antioxidant assays are usually required as an individual compound or group of compounds may exhibit different antioxidative powers on different assays (Thaipong, Boonprakob, Crosby, Cisneros‐Zevallos, & Hawkins Byrne, 2006). However, the results of our previous studies on Gac peel have shown that carotenoid extracts from Gac peel do not possess DHPH radical scavenging activity and also do not have significant activity on an iron reducing power assay. Only ABTS radical scavenging activity of the carotenoid extracts from Gac peel was found to be significant and that was highly correlated with the total carotenoid content in the extracts (Chuyen, Roach, Golding, Parks, & Nguyen, 2017a; Chuyen et al., 2017b). Thus, the ABTS radical scavenging activity was selected to represent the total antioxidant capacity of carotenoid extracts from Gac peel in this study.

The ABTS antioxidant assay of Gac peel extracts was carried out based on the methods described by Thaipong et al., 2006. The ABTS stock solution (7.4 mmol/L) and the potassium persulfate stock solution (2.6 mmol/L) were mixed with a ratio of 1:1 and left to react for 12–16 hr in a dark room. The ABTS working solution was then made by diluting the reacted solution with methanol to obtain an absorbance of 1.1 ± 0.02 units at 734 nm on a Cary 50 Bio UV‐Visible spectrophotometer (Varian Australia Pty. Ltd., Mulgrave, VIC, Australia).

A volume of 2.85 ml of the ABTS working solution and 0.15 ml of extract from Gac peel or 0.15 ml of standard Trolox solution were transferred into a test tube and the mixture reacted for 2 hr in a dark room. The absorbance of this reacted solution was then determined at 734 nm using the spectrophotometer. The ABTS antioxidant activity of the Gac peel extracts was expressed as μmole Trolox equivalents (TE) based on the standard curve of the Trolox solutions.

### 2.6 Statistical analysis

All experiments were repeated in triplicate and the results were expressed as the mean values ± standard deviations. The overall statistical significance for each experiment was determined using the analysis of variance test (ANOVA) and the LSD post‐hoc test was used for comparisons amongst the mean values if the ANOVA was significant. Differences were considered to be significant at *p* < .05.

## 3 RESULTS

### 3.1 Microwave assisted‐extraction

#### 3.1.1 Effect of microwave power on the temperature of the extract

Preliminary experiments showed that the continuous microwave radiation brought the extraction solvent to boil very quickly at all applied power levels (data not shown). Therefore, an intermittent microwave radiation with 30 s “on” and 30 s “off” alternatively was applied to prolong the extraction time. The temperature of 60°C was selected as upper limit for the extraction process because the results of published studies which have shown that carotenoids are severely degraded above this temperature (Fratianni, Cinquanta, & Panfili, 2010; Pasquet et al., 2011).

**Figure 2:**
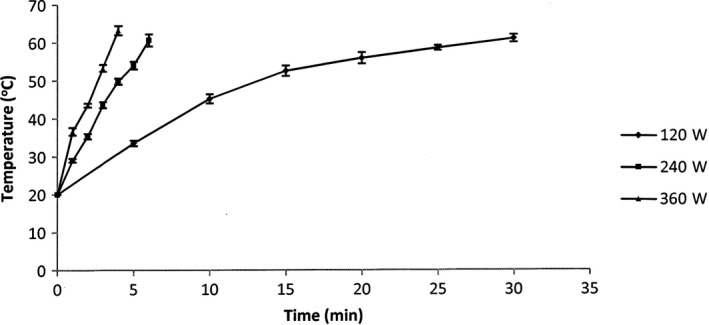
Change in temperature of the extracts at different microwave powers. The results are expressed as mean values, and the error bars show standard deviations of three replicates (*n* = 3)

The variation in the used microwave power led to a significant difference in the temperature increase in the extract (Figure 2). The temperature of the extraction at 360W rapidly increased from room temperature (20°C) to 63°C in 4 min while the MAE at 240W also reached 61°C after 6 min. When the microwave power was reduced to 120W, the temperature of the extract was retained below 60°C for 30 min (Figure 2). The determination of the absorbed power showed that the microwave energy absorbed by the extraction mixtures at 120W, 240W, and 360W was 0.32, 0.69 and 0.83 W/g, respectively. The high levels of microwave power absorbed at 240W and 360W were responsible for the rapid increase in the temperature of the extraction mixtures even the intermittent microwave radiation procedure has been applied. In contrast, the extraction at 120W could be maintained for a much longer time because its absorbed power was significant lower than that of the other extractions.

#### 3.1.2 Carotenoid extraction yield

**Figure 3:**
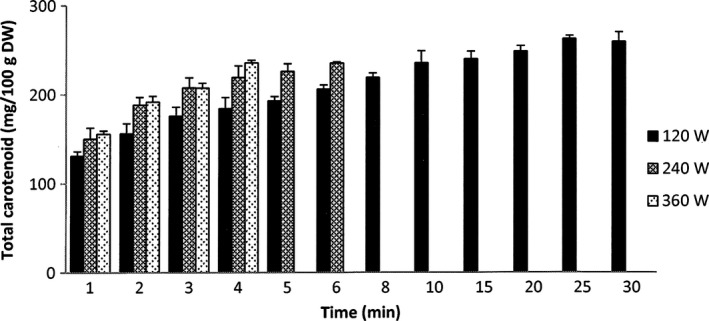
Carotenoid extraction yield of MAE at different microwave powers. The results are expressed as mean values, and the error bars show standard deviations of three replicates (*n* = 3)

The total carotenoid extraction yield of the MAE at 360W increased rapidly from 156 to 236 mg/100 g DW during 4 min of extraction time (Figure 3), which is comparable to a 6 min extraction and a 10 min extraction with microwave power at 240 W and 120 W, respectively. The extraction at 240W also caused a relatively high extraction yield of carotenoid (150 mg/100 g DW for 1 min) that rose steadily to 235 mg/100 g DW by the end of the process. For the extraction at 120W, the total carotenoid yield also slowly increased along with the slow increase in the temperature (Figure 3). The highest total carotenoid yield of this extraction (262 mg/100 g DW) was achieved at 25 min before being reduced slightly for the extended extraction time.

#### 3.1.3 Antioxidant capacity of the extracts

**Figure 4:**
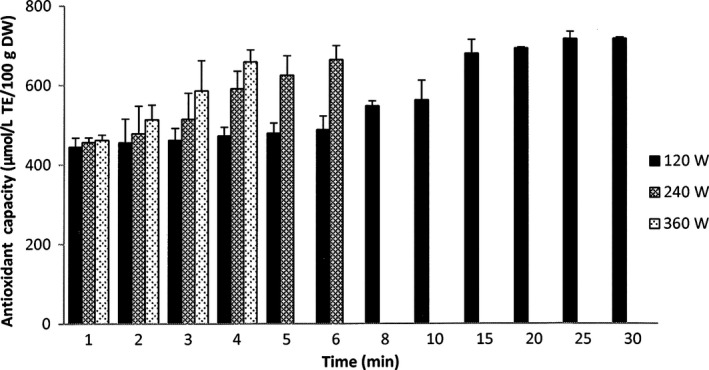
Antioxidant capacity of extracts from MAE at different microwave powers. The results are expressed as mean values, and the error bars show standard deviations of three replicates (*n* = 3)

The results of the different MAE extraction conditions on antioxidant capacity is presented in Figure 4 and shows that the antioxidant capacity of the extracts from Gac peel was similar in trend to that of the total carotenoid yield. The antioxidant capacity of the extract using 360 W of microwave power sharply increased to 659 μmol/L TE/100 g DW the end of the process, which was statistically comparable to the antioxidant values of 6 min extraction at 240 W (664 μmol/L TE/100 g DW) and 15 min extraction at 120 W (679 μmol/L TE/100 g DW). Although the antioxidant capacity of the extract at 120 W was lower than that of the other extractions when compared at correlative points of time, its maximum antioxidant yield (716 μmol/L TE/100 g DW at 25 min) was significantly higher than the maximum values of the others.

### 3.2 Ultrasound assisted‐extraction

#### 3.2.1 Carotenoid extraction yield

**Figure 5:**
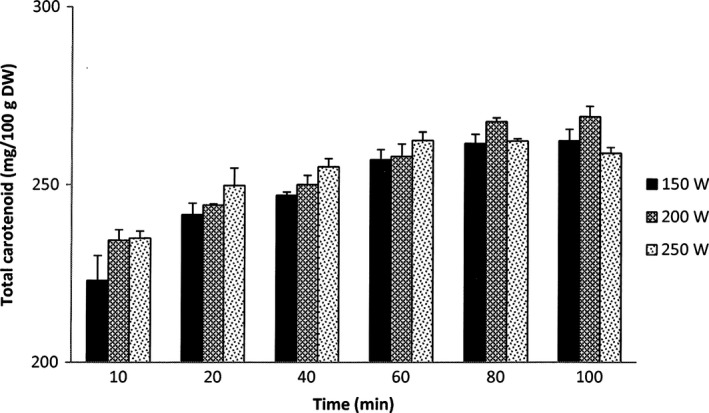
Carotenoid extraction yield of UAE at different powers. The results are expressed as mean values, and the error bars show standard deviations of three replicates (*n* = 3)

The results of the total carotenoid extraction yield of the UAE carried out at different ultrasonic powers (150, 200, and 250W) for different extraction times are presented in Figure 5. The results showed that carotenoid extraction yield from Gac peel gradually increased with the extraction time at all three ultrasound powers. For the extraction at 250W, the yield of carotenoid reached the highest level (263 mg/100 g DW) after one hour of extraction and slowly decreased with longer extraction times. The extractions at 150W and 200W resulted in the highest carotenoid yields at the extraction time from 80 to 100 min, which fluctuated in the ranges of 262 and 268 mg/100 g DW, respectively.

#### 3.2.2 Antioxidant capacity of the extracts

**Figure 6:**
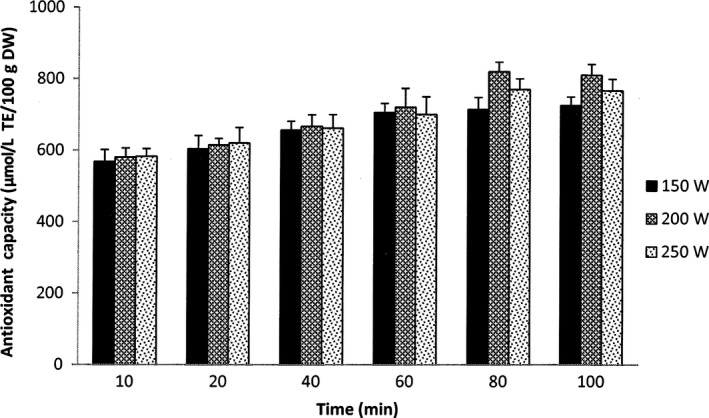
Antioxidant capacity of extracts from UAE at different powers. The results are expressed as mean values, and the error bars show standard deviations of three replicates (*n* = 3)

The results in Figure 6 show that a very high antioxidant capacity of the extracts (568–583 μmol/L TE/100 g DW) was achieved after only 10 min of extraction. The antioxidant capacity of the extracts then slowly increased along with the extraction time. However, there was no significant difference in the antioxidant capacity was found among the extracts obtained from the different ultrasonic powers until 80 min of extraction. After 80 min, the extractions at 200W and 250W obtained their highest levels of antioxidant capacity (820 and 770 μmol/L TE/100 g DW, respectively). When the extraction time extended to 100 min, a reduction in the obtained antioxidant capacity of those extractions was observed but the difference was not significant compared to that at 80 min. For the extraction at 150W, the antioxidant capacity of the extracts significantly increased until 60 min of the extraction. After 60 min, no significant improvement in the antioxidant capacity was observed.

## 4 DISCUSSION

In the microwave‐assisted extraction, the temperature of the extraction mixture increases by absorbing microwave energy. The increase in the temperature results in the lower viscosity of the solvent that promotes the diffusion rate of the desired compounds from the materials into the extraction medium (Eskilsson & Björklund, 2000). The heating using microwave energy also causes the rupture of the material cell walls which allows solvent to penetrate into the solid matrix to dissolve and release the compounds inside the cells into the liquid phase (Zhou & Liu, 2006). However, one of the obstacles of the MAE is the rapid increase in temperature of the extraction mixture that may terminate the extraction process quickly due to the boil of the solvent. When the extraction is terminated early, the desire compounds are not sufficiently diffused from the material into the solvent and consequently the extraction yield is reduced (Hiranvarachat & Devahastin, 2014; Nguyen et al., 2016). In this study, the use of intermittent microwave operation using 30 s “on” then 30 s “off” to cool down the extraction mixture could extend the extraction time significantly compared to the continuous microwave heating.

The significant longer extraction time of the MAE carried out at 120W resulted in the highest extraction yield of both total carotenoid and antioxidant capacity compared with the extractions carried out at 240W and 360W. However, the increase in the extraction yield was not proportional to the extraction time throughout the process. The results showed that the carotenoid extraction yield of the MAE at 120 W reached the highest level at 25 min of the extraction and was slightly lowered when the extraction was prolonged to 30 min. This reduction may be caused by the greater loss of carotenoids, the thermal sensitive compounds, at higher temperatures while the extraction rate was lowered due to the decrease in the solute concentration gradient between material and the solvent (Daood, Kapitány, Biacs, & Albrecht, 2006; Fratianni et al., 2010). This result is consistent the results found in previous studies on the extraction of carotenoids using microwave irradiation. For example, the highest extraction yield of fucoxanthin from microalgae was achieved after 5 min and then decreased in a MAE extraction for 15 min (Pasquet et al., 2011). The extraction time of 7.5 min was found as the best extraction time for recovering β‐carotene in a 15‐min MAE extraction of carrot peel (Hiranvarachat & Devahastin, 2014).

In comparison with a conventional extraction using the same batch of Gac peel sample and ratio of solvent to material (Chuyen et al., 2017c), the MAE at 120W obtained a lower carotenoid yield and a comparable antioxidant capacity yield (Table 1). However, the total extraction time of the MAE was sixfold shorter than that of the other (25 min compared with 150 min). Previous studies on extraction of carotenoids also showed that MAE resulted in higher carotenoid yields and shorter extraction times compared to conventional extraction methods. For example, MAE extraction of astaxanthin from *Haematococcus pluvialis* at 141 W for 5 min resulted in a higher extraction yield compared to the conventional stirring extraction for 12 hr (Zhao, Chen, Zhao, & Hu, 2009). Similar extraction efficiencies of carotenoids from a microalga (*Cylindrotheca closterium*) were also obtained with 5 min of MAE at 50W and 60 min of a conventional soaking extraction in acetone (Pasquet et al., 2011).

**Table 1:** A comparison of carotenoid and antioxidant capacity extractions from Gac peel using different extraction methods

Extraction method	MAE	UAE	CE*
Total carotenoid yield (mg/100 g DW)	262.3 ± 3.5^a^	267.7 ± 1.2^b^	271.1 ± 8.5^b^
Antioxidant capacity yield (μmol/L TE/100 g DW)	715.8 ± 18.1^a^	819.9 ± 26.5^b^	737.3 ± 23.8^a^
Extraction time (minute)	25	80	150
Energy consumption (kcal)	43	229	3.5

MAE, Microwave‐assisted extraction; UAE, Ultrasound‐assisted extraction; CE, Conventional extraction.

Values with same superscript in each row are not significantly different (*p* < .05).

*Source: Chuyen et al. (2017c).

For the ultrasound‐assisted extraction, the higher extraction yield of the UAE carried out at 200W compared to that carried out at 150W may be due to the greater cell wall disruption of Gac peel material when the higher ultrasonic power was applied (Chemat et al., 2017). However, the mechanism of the mass transfer based on the cell breakage is not adequate for explaining the reduction in the extraction yield resulted by the UAE carried out at 250W compared to that at 200W. The previous studies have found that lutein and β‐carotene, the major carotenoids in Gac peel (Chuyen et al., 2017a), were significantly degraded by ultrasound treatments and the degradation was greater with the increase in ultrasonic power (Sun, Ma, Ye, Kakuda, & Meng, 2010; Sun, Xu, & Godber, 2006). Thus, the greater loss of carotenoids caused by the UAE carried out at 250W may be a reason for its lower extraction yield compared to the UAE carried out at 200W.

The results of this study showed that after 80 min extraction, the UAE carried out at 200W obtained a carotenoid yield similar to that from 150 min using a conventional extraction with the same solvent‐to‐material ratio. However, the antioxidant capacity of this UAE was significantly higher than the maximum antioxidant capacity of the conventional method (Table 1). This result is in agreement with previous studies on UAE for phytochemical extraction which found that UAE can promote the release of not only carotenoids but also other bioactive compounds that contribute to the increase in the recovered antioxidant capacity (Abid et al., 2014; Chemat et al., 2017). Many other studies have also indicated that the application of ultrasound in the extraction of carotenoids can improve the extraction efficiency, enhance the antioxidant capacity of extracts and reduce the extraction time compared to the conventional extraction methods. For example, the extraction yield of lutein from egg yolk using UAE for 10 min was 4 times higher than the yield obtained from the conventional extraction with hexane for 20 min (Yue, Xu, Prinyawiwatkul, & King, 2006). Whilst UAE of β‐carotene from mandarin (*Citrus succosa* Hort) peel using ethanol resulted in a significantly higher extraction yields compared to the conventional extraction at all investigated extraction times, temperatures and ratios of solvent to material (Sun, Liu, Chen, Ye, & Yu, 2011). The extraction time for recovering β‐carotene from carrots (*Daucus carota*) was also shown to be reduced 3 times using UAE (Li, Fabiano‐Tixier, Tomao, Cravotto, & Chemat, 2013).

In comparison to the MAE extraction, the results in this study showed that UAE resulted in significantly higher extraction yields of both total carotenoid and antioxidant capacity (Table 1). This improved yield could be related to the greater amount of bioactive compounds being diffused into the solvent over a longer period. The lower thermal degradation of bioactive compounds caused by the UAE, which was carried out at 20°C, could have also contributed to its higher extraction efficiency compared with the MAE.

Although the ultrasound‐assisted extraction showed an improvement in the extraction efficiency compared to the microwave‐assisted extraction and the conventional extraction for carotenoids and antioxidant capacity from Gac peel, its energy consumption was much higher than that of the others (Table 1). The UAE using ultrasonic power of 200W for 80 min consumed 229 kcal while the MAE using 120W of microwave power for 25 min used 43 kcal and the power consumption conventional extraction using a magnetic stirrer for 150 min was only 3.5 kcal (Table 1). Therefore, to develop an economical and practical UAE method for recovering carotenoids from Gac peel, further studies for reducing the power consumption while still maintaining or improving the high extraction yield are necessary.

## 5 CONCLUSION

MAE and UAE at different power levels were investigated for the extraction of carotenoids and antioxidant capacity from Gac peel. The applied microwave and ultrasonic powers significantly increased the recovery of carotenoids from the peel and antioxidant capacity of the extracts. The UAE resulted in a greater antioxidant capacity extraction yield compared to the MAE and the conventional extraction. Although the MAE and UAE did not show any significant improvement in carotenoid extraction yield, the extraction time was significantly lower compared to the conventional extraction. The advantages of MAE and UAE for the extraction process of Gac peel in this experiment were obtained by investigating the extraction parameters in an individual manner. However, to maximize the extraction of carotenoids and antioxidant capacity from Gac peel, the interactive effects of the parameters should be studied and the determination of optimal conditions for MAE and UAE is recommended.

## CONFLICT OF INTEREST

The authors declare no conflicts of interest.
